# Enhancement of Emulsifying Activity in Soy-Protein-Based Products by Partial Substitution with Zein Hydrolysates and Transglutaminase Addition

**DOI:** 10.3390/foods14081353

**Published:** 2025-04-14

**Authors:** Zhihao Guo, Weiyu Li, Yuan Xue, Liying Bo, Jian Ren, Chunli Song

**Affiliations:** 1 College of Food and Bioengineering, Qiqihar University, Qiqihar 161006, China; guoxiaobaiy@163.com (Z.G.); 17604520971@163.com (W.L.); jmsdxxy@163.com (Y.X.); boliying1746@126.com (L.B.); renjian1970789@163.com (J.R.); 2 School of Educational Science, Jiamusi University, Jiamusi 154007, China

**Keywords:** SPI, zein, transglutaminase, cross-linking, emulsifying property

## Abstract

Partially substituting other proteins in soy-protein-based products is an effective method to meet nutritional and application requirements. However, the emulsifying properties of soybean protein isolates (SPI) when partially substituted with zein hydrolysates (ZH) remain unknown. In the present work, protein blend (0 h-SPI/ZH) from SPI and ZH with a ratio of 3.5: 1 (*w*/*w*) was treated by transglutaminase (TGase) for 0, 0.5, 1.0, and 1.5 h, respectively. SDS-PAGE analysis results indicate protein polymers were generated in SPI/ZH conjugates. Emulsifying activity of the conjugates (1.5 h-SPI/ZH) was significantly increased from 23.69 to 28.13 m^2^ g^−1^ in comparison with SPI, and there was no statistically significant difference (*p* < 0.05) in emulsion stability. The apparent viscosity, surface hydrophobicity of the SPI/ZH conjugates were significantly increased. Emulsion droplet size and zeta potential stabilized by 1.5 h-SPI/ZH were also increased; the values were 64.73 to 80.79 r.nm and −21.8 to −29.9 mV, respectively. CLSM results indicate that 1.5 h-SPI/ZH conjugates stabilized the emulsion and had a thicker adsorption layer. Overall, high values of negative zeta potential and suitable molecular weight distribution of the SPI/ZH conjugates might be responsible for the improved emulsifying property. This study provides insights for the preparation of soy-protein-based products as a promising food emulsifier.

## 1. Introduction

The functional properties of plant-based proteins are being extensively investigated due to growing consumer demand for novel plant-based products. Soy protein has demonstrated significant potential in stabilizing emulsions in plant-based food systems, due to its unique structural characteristics and amphiphilic properties [1]. However, SPI-stabilized emulsions face challenges in maintaining stability while balancing nutritional and cost considerations, prompting exploration of hybrid protein systems through partial substitution [2].

Zein, a corn-derived prolamin comprising 45–50% of corn protein [3] emerges as a strategic complement to SPI. Its amino acid profile is rich in amino acids, namely glutamine, proline, alanine, and leucine, making it nutritionally synergy with SPI composition [4]. Though zein’s hydrophobicity limits aqueous applications, enzymatic hydrolysis has enabled its functional deployment [5]. Zein hydrolysates (ZH) provide abundant glutamine groups (about 21.4%) [6] that serve as acyl donors in transglutaminase (TGase)-catalyzed cross-linking [7], while SPI contributes essential lysine residues as acyl acceptors. This biochemical complementarity establishes SPI/ZH conjugates as optimal substrates for TGase-mediated structural engineering. Crucially, the intrinsic lysine deficiency of ZH (<3% molar ratio) [5] fundamentally governs its cross-linking capacity. This compositional trait creates competitive inhibition during TGase-catalyzed reactions by preferentially directing lysine residues toward SPI-ZH conjugation rather than SPI self-cross-linking, thereby effectively suppressing excessively inter- or intramolecular cross-linking of SPI.

The cross-linking mechanism fundamentally alters protein functionality through controlled structural reorganization. TGase catalyzes covalent bonds between glutamine’s γ-carboxyamide and lysine’s ε-amino groups [7], inducing polypeptide chain unfolding and hydrophobic group exposure [8]. Moderate cross-linking enhances emulsion activity by optimizing interfacial adsorption and reducing tension [9,10]. However, to our knowledge, the potential benefits of zein substitution on the emulsifying and emulsion-stabilizing properties of soy-protein-based products generated by TGase addition have not been sufficiently studied or reported.

Therefore, this study aims to investigate the emulsifying properties and stabilization mechanisms of soy protein isolate (SPI)/zein hydrolysate (ZH) conjugates fabricated through transglutaminase (TGase)-mediated cross-linking. By partially substituting ZH with SPI and regulating TGase reaction times, we sought to use the complementary amino acid profiles of the two proteins and elucidate how enzymatic cross-linking modulates emulsion performance.

## 2. Materials and Methods

### 2.1. Materials and Chemicals

Soybean protein isolate (referred to as SPI) was extracted from defatted soybean meal Harbin Binxian Yuwang Vegetable Protein Co., Ltd. (Harbin, China) following the method described by our previous study [11], and the protein content was 90.3%. Corn gluten meal powder obtained from Cofco Biotechnology Co., Ltd. (Qiqihar, China); TGase with an enzyme activity of 3000 U/g provided by Jiangsu Yiming Fine Chemical Co., Ltd. (Taixing, China); Low-molecular-weight standard proteins, including blue dextran 2000, Trypsin inhibitor, Bacillus subtilis, oxidized glutathione, and reduced glutathione, were supplied by Beijing Xinjingke Biotechnology Co., Ltd (Beijing, China).

### 2.2. Experimental Methods

#### 2.2.1. Preparation of ZH

The preparation of zein hydrolysates (12.0% hydrolysis degree, referred to as ZH) was carried out following the method described by Zheng et al. [12]. Briefly, the corn gluten meal was initially crushed into a fine powder and then sieved through an 80-mesh screen. The resulting powder was then subjected to acetone extraction to remove most pigments, followed by centrifugation for 15 min to collect the precipitates. The precipitates were mixed with 70% ethanol at 60 °C for 2 h, then centrifuged to obtain the supernatant. This ethanol extraction procedure was repeated two additional times with the remaining precipitates. All supernatants were combined and evaporated at 60 °C until reduced to one-quarter of their original volume. After evaporation, the sample was diluted with cold water without agitation. The resulting precipitates were collected by centrifugation and subsequently freeze-dried under a vacuum.

#### 2.2.2. Preparation of the SPI/ZH Conjugates from SPI and ZH with TGase Addition

The prepared SPI was thoroughly swelled at 4 °C for 5 h and then dispersed at pH 7.5 with a protein concentration of 60 mg·mL^−1^. Then, it was heated at 90 °C for 5 min and cooled down to room temperature. A mixture of SPI and ZH in a mass ratio of 3.5:1 (*w*/*w*) was prepared by the addition of ZH dispersion with a protein final concentration of 40 mg·mL^−1^ at pH 7.5. Subsequently, TGase enzyme was added at 10 U/g protein. After incubation at 45 °C for 0.5, 1, and 1.5 h, respectively, the samples underwent heat treatment in a water bath at 85 °C for 5 min and then cooled to room temperature. The resulting samples after being lyophilized were denoted as follows: 0 h-SPI/ZH, 0.5 h-SPI/ZH, 1 h-SPI/ZH, and 1.5 h-SPI/ZH.

#### 2.2.3. SDS-PAGE

SDS-PAGE was performed using 12% separating gels and 4% stacking gels to determine the molecular weight distribution of the prepared SPI/ZH conjugates. The SPI/ZH conjugates samples with a concentration of 3 mg·mL^−1^ was loaded with 12 µL. β-mercaptoethanol was added to prepare the reducing buffer for electrophoresis samples. After electrophoresis, the gel was subjected to protein staining and destaining treatment.

#### 2.2.4. Measurement of Free Amino Group Contents

The content of the free amino groups was determined by the method of Li [13]. To prepare the OPA solution, 2.0 g of sodium dodecyl sulfate was dissolved in 30 mL of 0.4 mol/L sodium borate buffer (pH 9.5). Next, 200 µL of β-mercaptoethanol, 80 mg of OPA in 1 mL of ethanol (ETOH), and the borate buffer were combined to obtain a final solution volume of 100 mL. A mixture of 3 mL of the OPA reagent and 3 mL of the sample solution was allowed to stand for 5 min before measuring the absorbance at 340 nm using a UV-2401PC spectrophotometer (Shimadzu, Kyoto, Japan). Leucine solutions (0–36 µg/mL) were utilized to generate a standard curve, and the results were reported as -NH_2_ mol·kg^−1^ protein. The grafting degree (DG) was calculated according to Equation (1):

(1) $$\mathrm{DG}\left(\%\right)=\frac{C_{0}-C_{1}}{C_{0}}\times 100$$

where C_0_ is the free amino groups contents of the sample (0 h-SPI/ZH) before modification, mol·kg^−1^;

C_1_ is the free amino group content of the SPI/ZH conjugates (0.5 h-SPI/ZH, 1 h-SPI/ZH, 1.5 h-SPI/ZH), mol·kg^−1^.

#### 2.2.5. Fourier-Transform Infrared Spectroscopy (FT-IR) Analysis

The potassium bromide tablet method [11] was employed for infrared spectral analysis. The experimental procedure was as follows: 2 mg of the samples and 200 mg of potassium bromide were placed in a mortar, where the samples were ground into a uniform powder and pressed into thin discs. All FTIR spectra were obtained using a Spectrum One FTIR spectrometer (Perkin Elmer Inc., Norwalk, CT, USA) by scanning from 4000 to 400 cm^−1^ at a resolution of 1 cm^−1^. A total of 32 scans were used.

#### 2.2.6. Solubility

The protein samples were accurately weighed to 20 mg (on a protein basis), and 10 mL of pH 7.5 PBS buffer solution was added to each. After swirling and mixing for 30 s, the samples were refrigerated at 4 °C overnight to ensure complete hydration. The protein content was determined using the Folin-phenol method after collecting the supernatant and centrifuging at 10,000 rpm at 4 °C for 10 min. The percentage of the protein content in the supernatant to that of the initial total protein in the sample indicates its solubility [14].

#### 2.2.7. Analysis of Emulsifying Properties

The emulsifying properties of the protein samples were determined according to the method described by Li et al. [15] for the assessment of protein-emulsifying activity and emulsion stability, 150 mL of protein sample dispersion with a concentration of 1 mg·mL^−1^ (pH 7.5) was mixed with 50 mL of refined soybean oil, followed by homogenize using a high-speed homogenizer at 12,000 rpm for 1 min (T25, IKA-Works GmbH & Co. KG, Staufen, Germany). At both 0 min and 10 min after homogenization, 50 µL of the prepared emulsion was quickly extracted from the bottom and transferred into a solution containing 5 mL of SDS at a concentration of 1 mg·mL^−1^. After shaking, the absorbance value of the resulting emulsion at a wavelength of 500 nm (RF-5301PC Shimadzu, Kyoto, Japan) was measured. The Emulsifying Activity Index (EAI) and Emulsion Stability Index (ESI) can be calculated as follows (Equations (2) and (3)):

(2) $$\mathrm{EAI}\;(m^{2}\cdot g^{-1})=\frac{2\times 2.303\times A_{0}\times \mathrm{Dilution}\;\mathrm{ratio}}{C\times (1-\varphi )\times 10^{4}}$$

(3) $$\mathrm{ESI}\left(\%\right)=\frac{A_{10}}{A_{0}}\times 100$$

where A_0_ is the light absorption value 0 min after the end of homogenization;

C is the concentration of the protein sample solution (g·mL^−1^);

ϕ is 0.25 (the oil volume fraction);

A_10_ is the absorbance value after 10 min of homogenization.

#### 2.2.8. Droplet Size Distribution and Zeta Potential of the Prepared Emulsions

Droplet size (r.nm) and zeta potential of the prepared emulsion were measured by dynamic light scattering (DLS) using a Nano-ZS90 particle size analyzer (Malvern Panalytical Ltd., Malvern, UK) at 20 °C. To determine the droplet size, the protein emulsion was diluted to a concentration of 0.1 mg·mL^−1^ using 20 mg·mL^−1^ sodium dodecyl sulphate (SDS) solution at pH 7.5. The instrument operated in a back-scattering configuration, with detection at a 173° scattering angle using an avalanche photodiode and a helium-neon laser (λ = 633 nm). The refractive indices for soy oil and water were 1.47 and 1.33, respectively. Droplet size was measured as the Z-average (r.nm). Zeta-potential was analyzed at a scattering angle of 13° and a temperature of 20 °C.

#### 2.2.9. Measurement of Surface Hydrophobicity

The surface hydrophobicity of proteins was assessed using the ANS (1-aniline-8-naphthalene sulfonic acid) fluorescent probe method. Following centrifugation of the dispersion at 10,000 rpm for 15 min, 4 mL of the supernatant (with a protein concentration ranging from 0.025 to 0.4 g·L^−1^ and a pH of 7.5) was collected and combined with 20 µL of ANS at a concentration of 8 mmol·L^−1^. This mixture was then incubated in the dark for 15 min, with phosphate buffers (pH 7.5) serving as controls. The relative fluorescence intensity measurement conditions included an excitation wavelength of 390 nm, emission wavelength of 470 nm, and slit width set at 5 nm. A graph plotting protein concentration on the *x*-axis against relative fluorescence intensity on the *y*-axis revealed that the slope representing the linear relationship between these two parameters corresponded to the surface hydrophobicity value.

#### 2.2.10. Apparent Viscosity of SPI and Its Modified Products Dispersions

The apparent viscosity of SPI and SPI/ZH conjugates dispersions was determined using a Malvern rheometer (Kinexus Pro^+^ Malvern Instrument, Malvern, UK) [16]. Protein sample dispersion with a pH of 7.5 and a concentration of 40 g·L^−1^ was prepared. During measurement, approximately 1 mL of the sample was slowly dispensed onto the fixture, a cone plate with a diameter of 60 mm and a cone angle of 0.5° was selected, and the gap value was set as 1 mm during the measurements. The apparent viscosity of the sample was measured at a temperature of 25 °C and frequencies ranging from 0.1 to 100 s^−1^.

#### 2.2.11. Confocal Laser Scanning Microscopy (CLSM)

A specific volume of protein solution was mixed with refined soybean oil, and after pre-emulsification at room temperature (10,000 rpm, 2 min), the emulsion was homogenized using a high-pressure homogenizer for 2 min (20 MPa) to produce the emulsion, with protein content and oil content of 0.5% (*w*/*v*) and 10% (*v*/*v*), respectively. Following the method by Zhang et al., laser confocal microscopy (CLSM; model SP8 DIVE, Leica Microsystems Inc., Wilmington, DE, USA) was employed for direct observation of the emulsion’s microstructure [17]. Before imaging, the prepared emulsion (1 mL) was mixed with 40 μL of a 0.01% *w*/*w* Nile Red and 0.1% *w*/*w* Nile Blue solution. The stained emulsions were immediately placed into a well slide. Fluorescence microscopy observations were made using a 40× oil lens to observe regions and layers, selecting Ar ions at 488 nm (Nile Red, oil phase dye) and He/Ne ions at 633 nm (Nile Blue, saccharide and protein dye) for laser pre-scanning in order to capture fluorescence images at a scanning density of 1024 × 1024 pixels. Subsequently, the images were processed using LAS X 3.5.6 software (Leica Microsystems CMS GmbH, Wetzlar, Germany).

## 3. Results and Discussion

### 3.1. Analysis of Polyacrylamide Gel Electrophoresis (SDS-PAGE)

**Figure 1:**
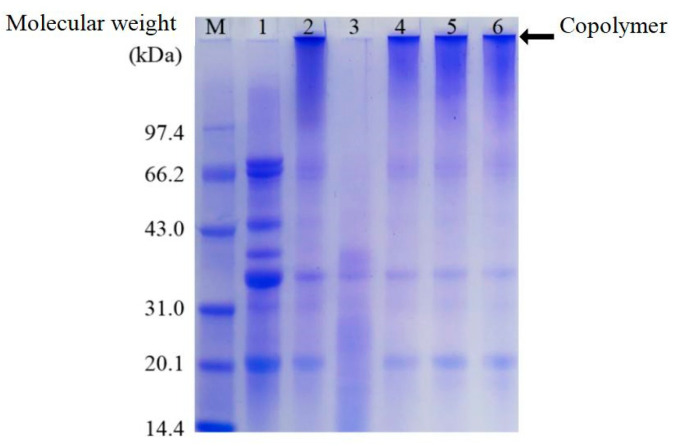
SDS–PAGE gels stained by Coomassie brilliant blue for proteins. Lane M, protein markers; Lane 1, soy protein isolates (SPI); Lane 2, SPI with TGase addition for 1.5 h; Lanes 3–6, SPI-zein hydrolysates (ZH) blend with TGase addition for 0, 0.5, 1.0, and 1.5 h (0 h-SPI/ZH, 0.5 h-SPI/ZH, 1 h-SPI/ZH, and 1.5 h-SPI/ZH), respectively.

SDS-PAGE, along with Coomassie Brilliant Blue staining, was used to illustrate the distribution of molecular weights in SPI and SPI/ZH conjugates. As illustrated in Figure 1, the polymers were generated in 0.5 h-SPI/ZH, 1 h-SPI/ZH, and 1.5 h-SPI/ZH products (Figure 1, lanes 4–6), the phenomenon was similar to the cross-linked SPI (Figure 1, lane 2). On one hand, TGase-induced SPI self-cross-linking contributed to the polymer generation [18,19]. On the other hand, significantly decreased bands in ZH were observed. Both amino acid compositional traits of the two proteins (SPI and ZH) [5,6] and smaller steric hindrance of ZH competitively inhibit SPI self-cross-linking during TGase-catalyzed reactions. Therefore, ZH could also conjugate to the polymer. From SDS-PAGE results, we deduced that SPI/ZH conjugates contained polymer. Hence, the prepared conjugates might contain partly unreacted ZH hydrolysates.

### 3.2. FT-IR Analysis

**Figure 2:**
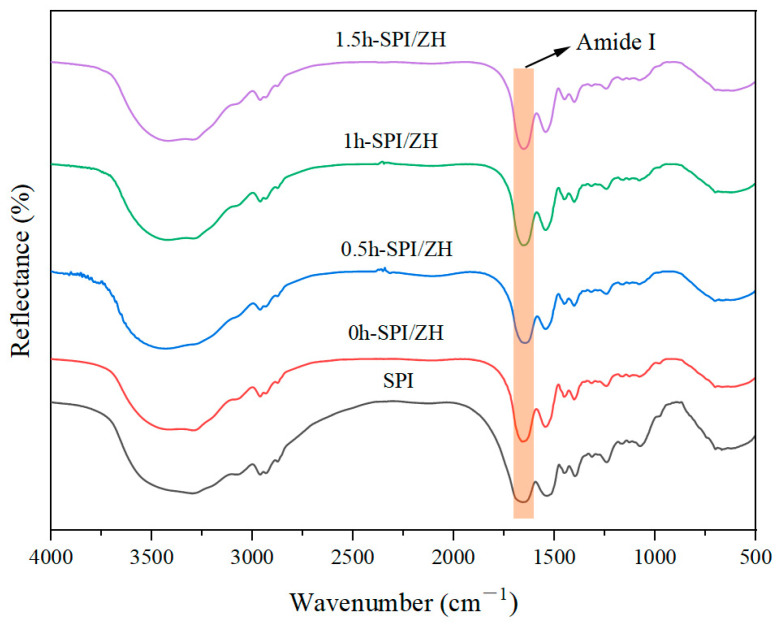
FTIR spectra of the soybean protein isolates (SPI) and SPI/ZH conjugates. The products of SPI-zein hydrolysates (ZH) blend with TGase addition for 0, 0.5, 1.0, and 1.5 h were named 0 h-SPI/ZH, 0.5 h-SPI/ZH, 1 h-SPI/ZH, and 1.5 h-SPI/ZH, respectively.

FT-IR analysis of SPI and SPI-MD conjugates is shown in Figure 2. The amide I band (1600–1700 cm^−1^) in the protein’s characteristic absorption spectrum reflects its secondary structures. Wavenumbers 1650–1660 cm^−1^, 1610–1640 cm^−1^, 1660–1700 cm^−1^, and 1640–1650 cm^−1^ were assigned to the α-helix, β-sheet, β-turn, and random coil, respectively [20]. The secondary structure results of both native SPI and SPI-MD conjugates are listed in Table 1. Curve fitting was conducted using PeakFit 4.12 to calculate the area percentage of each structural component within the main absorption peak, allowing for the determination of the relative content of each secondary structural unit (Table 1).

As shown in Table 1, the 0 h-SPI/ZH conjugate exhibited increased α-helix and β-sheet contents compared to SPI. In contrast, the β-turn content demonstrated an inverse trend, with a gradual reduction observed in 0 h-SPI/ZH relative to SPI. These structural alterations align with previous findings reported by Peng et al. [20]. The 0.5 h-SPI/ZH sample, subjected to a shorter cross-linking duration, showed no significant changes in secondary structure composition compared to 0 h-SPI/ZH. However, prolonged cross-linking time resulted in an overall decreasing trend in α-helix, β-sheet, and random coil contents, consistent with the results of Mizuno et al. [21]. These results indicate that the TGase-mediated cross-linking process induced a more randomized secondary structure configuration in SPI/ZH conjugates compared to the non-cross-linked 0 h-SPI/ZH. Proteins with flexible structures that can open and quickly adsorb are beneficial for enhancing the functional properties of the cross-linked SPI/ZH conjugates.

### 3.3. Alterations in Solubility, Emulsion Droplet Size, and Zeta Potential

As shown in Table 2, the grafting degree (DG) of SPI/ZH conjugates over time (0.5–1.5 h) were 17.38%, 19.86% and 23.51%, respectively, which is consistent with the results obtained from SDS-PAGE. The solubility of protein blends and the SPI/ZH conjugates was increased, and protein blends reached a maximum value of 59.4%. This enhancement may be attributed to ZH addition, which has higher solubility. However, the solubility of the SPI/ZH conjugates gradually decreased with extended TGase treatment time. After 1.5 h of TGase treatment, the solubility decreased to 55.7%, although it remained higher than that of SPI, which has a solubility of 53.9%. This reduction can be attributed to the TGase-induced cross-linking generated protein copolymers, which reduced the solubility of the resulting products [22]. A suitable solubility (minimum of ~50%) is necessary to contribute to form a stable, elastic film at the interface, thus preventing coalescence at the oil/water interface caused by highly insoluble proteins [23].

The absolute zeta potential value indicates the stability of the system. When the zeta potential of a colloidal system is higher than 10 mV (absolute value), the system tends to be more stable [2]. As shown in Table 2, SPI exhibited an absolute zeta potential of 21.8 mV (absolute value). After 1.5 h of TGase treatment, the absolute zeta potential of SPI/ZH conjugates significantly increased to 29.9 mV (absolute value). This elevation may result from cross-linking reactions between lysine and glutamine residues, which lead to an enhanced surface charge density. The larger the absolute value of the zeta potential, the greater the surface charge of SPI and ZH. In this case, electrostatic repulsion can help maintain the stability of the system. Similar conclusions were drawn by Liu et al. [2] regarding the TGase cross-linked soy protein isolate–whey protein polymer. Additionally, the size of emulsion droplets exhibited a significant increase following the incorporation of ZH and TGase-mediated cross-linking. After 1.5 h of TGase treatment, the droplet size of SPI/ZH conjugates increased from 64.73 r.nm (SPI) to 80.79 r.nm. This phenomenon can be attributed to the formation of macromolecular polymers through TGase-catalyzed cross-linking between SPI and ZH, which promotes the generation of larger emulsion droplets and consequently enhances emulsifying activity [24].

### 3.4. Emulsification

**Figure 3:**
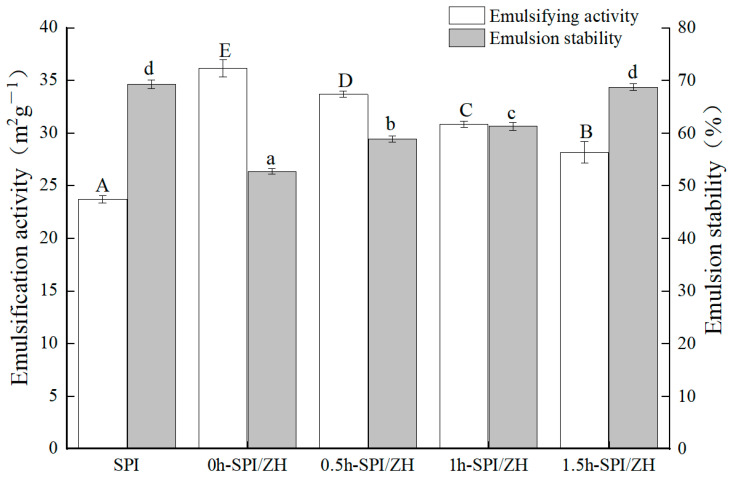
The emulsifying activity and emulsion stability of the soybean protein isolates (SPI) and SPI/ZH conjugates. The products of SPI-zein hydrolysates (ZH) blend with TGase addition for 0, 0.5, 1.0, and 1.5 h were named 0 h-SPI/ZH, 0.5 h-SPI/ZH, 1 h-SPI/ZH, and 1.5 h-SPI/ZH, respectively. Different letters show significant differences among results (*p* < 0.05).

The emulsification activity index (EAI) and emulsion stability index (ESI) are indicative of the proteins’ capacity to form emulsions by adsorption at the oil–water interface and their ability to maintain stability at the interface [25]. The emulsification activity and stability of SPI and its SPI/ZH conjugates were assessed using turbidimetry, with results presented in Figure 3.

As illustrated in Figure 3, the emulsification activity of the SPI/ZH conjugates was significantly higher than that of SPI. This improvement can be attributed to the increased solubility of the SPI/ZH conjugates following the incorporation of ZH, which leads to a reduction in interfacial tension and a subsequent enhancement in emulsifying activity. Based on a comprehensive analysis of emulsification activity and stability, it can be concluded that partial substitution of SPI with ZH after TGase treatment can enhance the emulsification activity of SPI but has no statistically significant impact on its emulsion stability.

### 3.5. Surface Hydrophobicity

The surface hydrophobicity is a key structural characteristic of proteins, providing insight into the protein surface in polar environments, and its relation to the structure, stability, and functionality of proteins [26]. The results for the surface hydrophobicity of SPI and SPI/ZH conjugates are presented in Table 2.

According to Table 2, the surface hydrophobicity of SPI increased with the addition of a hydrophobic protein (ZH) (53.53 vs. 57.31 in SPI and its blends), attributed to the high content of hydrophobic groups in ZH [27]. Subsequently, the surface hydrophobicity of the protein dispersions increases with increasing TGase reaction time in the range of 0.5–1.5 h and 1.5 h-SPI/ZH showed the highest surface hydrophobicity (72.21). This can be attributed to cross-linking reactions causing gradual embedding of hydrophilic groups within protein molecules and exposure of more hydrophobic residues on molecular surfaces, thereby enhancing oil–water interface activity and protein adsorption capacity [28]. Consequently, SPI and ZH blends following TGase cross-linking modification, both the surface hydrophobicity and emulsification activity of the generated conjugates were enhanced [29]. This results in increased surface hydrophobicity and flexibility, which enhances its functional properties [26].

### 3.6. Apparent Viscosity of Protein Dispersions

**Figure 4:**
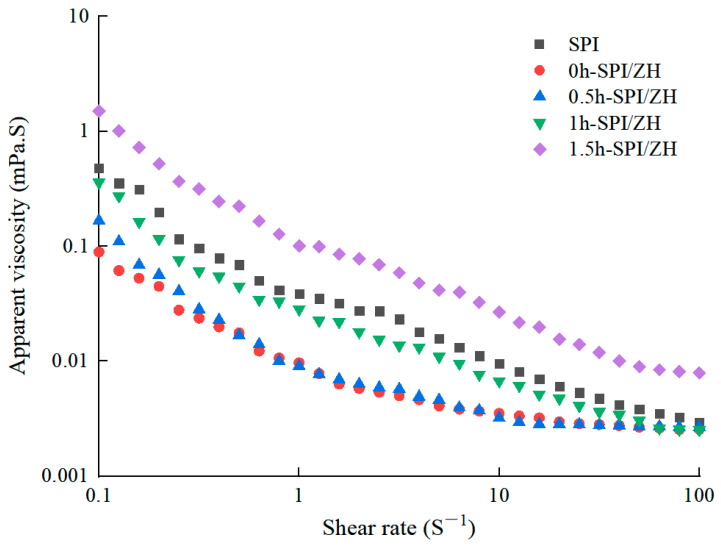
Apparent viscosity of the soybean protein isolates (SPI) and SPI/ZH conjugates. The products of SPI-zein hydrolysates (ZH) blend with TGase addition for 0, 0.5, 1.0, and 1.5 h were named 0 h-SPI/ZH, 0.5 h-SPI/ZH, 1 h-SPI/ZH, and 1.5 h-SPI/ZH, respectively.

The apparent viscosity determination results of the sample dispersions of SPI and SPI/ZH conjugates at a shear rate of 0.1–100 s^−1^ are depicted in Figure 4. As shown in Figure 4, the apparent viscosity of all test samples decreases with increasing shear rates within the range of 1 to 100 s^−1^, indicating a shear thinning phenomenon, characteristic of a pseudoplastic fluid [30]. The apparent viscosity order of the samples from lowest to highest was as follows: 0 h-SPI/ZH < 0.5 h-SPI/ZH < 1 h-SPI/ZH < SPI < 1.5 h-SPI/ZH. In contrast to protein blends, SPI/ZH conjugates exhibited an inclination towards increased apparent viscosity due to cross-linking-induced macromolecular polymer formation that elevates relative molecular mass of protein molecules and apparent viscosity in protein-like solutions [31]. The gradual improvement in emulsion stability over time (0 h–1.5 h) is partly due to an increase in viscoelasticity at the interface film, resulting in steric hindrance that prevents droplet aggregation [32], thereby enhancing emulsion stability.

### 3.7. Adsorbed Proteins

**Figure 5:**
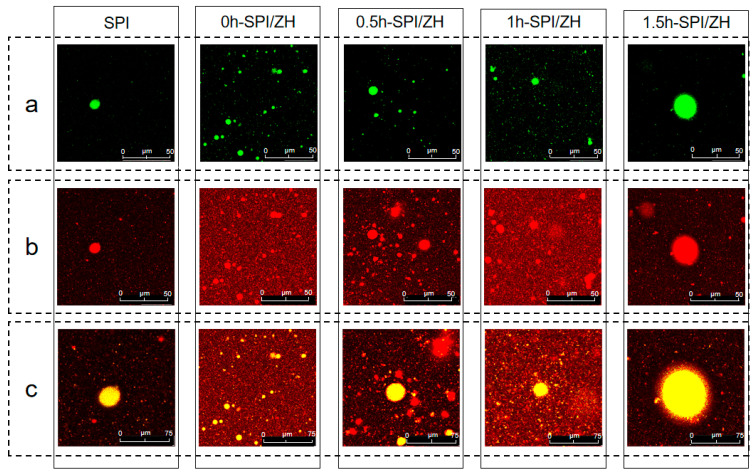
CLSM images of the soybean protein isolates (SPI) and SPI/ZH conjugates. The products of SPI-zein hydrolysates (ZH) blend with TGase addition for 0, 0.5, 1.0, and 1.5 h were named 0 h-SPI/ZH, 0.5 h-SPI/ZH, 1 h-SPI/ZH, and 1.5 h-SPI/ZH, respectively (**a**); Oil phase in SPI and SPI/ZH conjugates with different time; Protein phase in SPI and SPI/ZH conjugates with different time (**b**); Images of the combination of protein and oil phase in SPI and SPI/ZH conjugates with different time (**c**). The scale for (**a**,**b**) is 50 μm, while the scale for (**c**) is 75 μm. Protein was stained by Nile Blue (green) and oil was stained by Nile Red (red).

Confocal Laser Scanning Microscopy (CLSM) allows for the detailed observation of the microstructure of emulsions, thereby facilitating a deeper analysis of the mechanisms involved in protein emulsification and stabilization. Figure 5 presents the CLSM images of emulsions stabilized by SPI and SPI/ZH conjugates. The bright green regions represent the oil phase stained with Nile Blue (Figure 5a), while the red regions indicate protein-rich areas labeled with Nile Red (Figure 5b). Figure 5c illustrates the combined images of the protein and oil phase distributions.

From Figure 5, it can be observed that the emulsion droplets stabilized by SPI and SPI/ZH conjugate derivatives contained an oil phase in their core, with proteins localized at the droplet surface. This indicates that both SPI and its conjugates form water-in-oil (W/O)-type emulsions. As illustrated in Figure 5a, the weak oil-phase signal in the SPI-stabilized emulsion indicates minimal fat aggregation. Additionally, Figure 5c demonstrates a protein layer encapsulating the emulsion droplets. Compared to emulsion stabilized by SPI, emulsion stabilized by the SPI/ZH conjugates exhibited stronger fluorescent signals in the protein layer. Figure 5c demonstrates a uniform thickness and the highest fluorescence intensity in the adsorbed protein layer surrounding the droplets. This indicates that the highest protein adsorption concentration occurs in the 1.5 h-SPI/ZH sample. In addition, Figure 5c shows that the emulsion droplet size of the 1.5 h-SPI/ZH sample is the largest. This indicates that large molecular polymers are formed through TGase-catalyzed cross-linking between SPI and ZH, which is consistent with the previous results from DLS measurements.

As illustrated in Figure 5b, the number of protein-labeled droplets gradually decreases as the cross-linking reaction progresses, reaching its lowest level in 1.5 h-SPI/ZH. This trend is similar to the protein-phase signal intensity observed for SPI. The CLSM analysis indicates that the improved emulsification activity of SPI-ZH conjugates, following TGase-mediated cross-linking, is due to the creation of a biphasic system with suitable molecular weight distribution. In this system, protein molecules adsorb at the oil–water interface, forming a cohesive interfacial film that encapsulates the oil droplets [27].

## 4. Conclusions

This study demonstrates that emulsifying activity in soy protein was enhanced by partial substitution with zein hydrolysates and transglutaminase addition, which resulted in a significant increase in the emulsifying activity (from 23.69 m^2^·g^−1^ in SPI to 28.13 m^2^·g^−1^ in 1.5 h-SPI/ZH), as well as the emulsion stability. The underlying mechanisms are likely attributed to the enhanced water solubility, increased surface hydrophobicity (from 53.53 in SPI to 72.21 in 1.5 h-SPI/ZH), higher zeta potential (from −21.8 in SPI to −29.9 in 1.5 h-SPI/ZH), and the flexible structure of SPI/ZH conjugates. The cross-linking between SPI and ZH catalyzed by TGase forms protein macromolecular polymers, which enhances the adsorption of proteins at the oil–water interface and promotes the formation of larger emulsion droplets. At the same time, it creates a cohesive biphasic interfacial film that encapsulates the oil droplets. Furthermore, the interfacial-enhanced viscoelasticity provides spatial hindrance for the aggregation of oil droplets, thereby enhancing the emulsifying activity. However, the contribution of self-cross-linking of SPI induced by TGase remains unclear and requires further investigation. This research supports zein hydrolysates as a promising additive for improving food emulsification in the SPI-based food system.

## Figures and Tables

**Table 1:** The secondary structure composition of soy protein isolates (SPI), SPI-zein hydrolysates (ZH) blend with TGase addition for 0, 0.5, 1.0, and 1.5 h, respectively.

Samples	α-Helix (%)	β-Sheet (%)	β-Turn (%)	Random Coil (%)
SPI	13.35	37.1	36.68	13.54
0 h-SPI/ZH	14.28	42.34	30.17	13.21
0.5 h-SPI/ZH	14.28	42.34	30.17	13.21
1 h-SPI/ZH	13.04	37.25	36.32	13.39
1.5 h-SPI/ZH	10.87	27	40.21	11.04

**Table 2:** The grafting degree (DG), solubility, emulsion drop size, zeta potential, and surface hydrophobicity of soy protein isolate (SPI), SPI-zein hydrolysates (ZH) blend with TGase addition for 0, 0.5, 1.0 and 1.5 h, respectively.

Index	SPI	0 h-SPI/ZH	0.5 h-SPI/ZH	1 h-SPI/ZH	1.5 h-SPI/ZH
Grafting degree (%)	0	0	17.38 ± 0.20 ^c^	19.86 ± 0.28 ^b^	23.51 ± 0.32 ^a^
Solubility (pH 7.5)	53.9 ± 2.91 ^b^	59.4 ± 1.81 ^a^	57.6 ± 1.61 ^ab^	56.5 ± 0.90 ^ab^	55.7 ± 2.21 ^ab^
Emulsion drop size (r.nm)	64.73 ± 2.5 ^a^	74.87 ± 1.4 ^b^	78.01 ± 2.2 ^c^	80.22 ± 2.9 ^d^	80.79 ± 1.6 ^e^
Zeta potential (mV)	−21.8 ± 0.8 ^b^	−18.3 ± 0.7 ^a^	−25.9 ± 1.2 ^c^	−26.8 ± 1.1 ^c^	−29.9 ± 0.9 ^d^
Surface hydrophobicity	53.53 ± 0.32 ^a^	57.31 ± 0.26 ^b^	60.13 ± 0.92 ^c^	66.66 ± 0.34 ^d^	72.21 ± 0.17 ^e^

Different letters within the same row indicate that the means are significantly different according to one-way ANOVA analysis (*p* < 0.05).

## Data Availability

The original contributions presented in the study are included in the article; further inquiries can be directed to the corresponding author.
